# Concept and techniques for minimal invasive restorative dentistry: A review

**DOI:** 10.6026/973206300220522

**Published:** 2026-01-31

**Authors:** Ameer Akhil Ahmed Shaik, Pratik Surana

**Affiliations:** 1 Department of Dental Surgery and General Dentistry, Narayana Dental College, Nellore, Andhra Pradesh, India; 2 Department of Pedodontics and Preventive Dentistry, Maitri College of Dentistry and Research Centre, Durg, Chhattisgarh, India

**Keywords:** Minimal invasive dentistry, preventive dentistry, restorative technique

## Abstract

Minimal invasive restorative dentistry focuses on preserving healthy tooth structure while restoring damaged teeth. Emphasizing prevention, early diagnosis and less aggressive interventions, it aims to enhance both function and aesthetics. Therefore, it is of interest to review the principles, current methodologies and benefits, underscoring the importance of a patient centered approach in modern dental practice, ultimately promoting better oral health outcomes and patient satisfaction.

## Background:

Recent advancements highlight a shift toward minimally invasive techniques that conserve healthy tooth structure while achieving optimal clinical outcomes [1]. Traditionally, restorative dentistry often required extensive removal of healthy tooth material to fit restorations like dental crowns and bridges. This conventional approach has several drawbacks. The loss of healthy tooth structure can lead to increased vulnerability to future dental complications, such as decay or structural failure [1, 2]. Additionally, more invasive procedures can prolong the overall treatment time and impact the patient's experience [2]. In response to these challenges, minimally invasive techniques have emerged as progressive alternatives in restorative dentistry [3]. In response to the challenges posed by traditional restorative techniques, minimally invasive dentistry has gained prominence as a progressive alternative in restorative practices. These approaches prioritize the conservation of natural tooth structure and utilize advanced technologies and materials to achieve effective repairs [4]. Methods such as Silver Diamine Fluoride (SDF) and resin infiltration techniques are particularly noteworthy. SDF can help halt the progression of carious lesions, allowing for the preservation of tooth structure without the need for extensive drilling [5]. Meanwhile, resin infiltration is designed to penetrate early carious lesions, effectively sealing them and reinforcing the tooth while maintaining its integrity [6]. Biomimetic restorations have also emerged as a key aspect of minimally invasive dentistry, focusing on replicating the natural properties of teeth. This approach utilizes materials and techniques that mimic the mechanical and optical characteristics of natural tooth structure, enhancing both function and aesthetic [7]. Additionally, the use of Smart Burs, which are specially designed dental burs that remove decayed tooth material while preserving healthier tissue, further, exemplifies the shift towards conservative practices [8]. Therefore, it is of interest to thoroughly explore various facets of minimally invasive restorative techniques.

## Concept of minimal invasive dentistry:

According to Golden Triangle of Minimally Invasive Restorative Dentistry consists of three interconnected components: histopathology of tissue and disease, biomaterials and clinical handling. Each of these elements plays a crucial role in achieving successful outcomes with minimally invasive techniques [9].

## Histopathology of tissue/disease:

Understanding the histopathology of dental tissues and their associated diseases is fundamental to minimally invasive dentistry. This component involves studying the microscopic structure and changes in dental tissues affected by caries, trauma, or other conditions.

## Mineral content:

Healthy dental tissues contain minerals such as hydroxyapatite, which are crucial for maintaining tooth strength. In carious lesions, the mineral content is lost, leading to demineralization. By identifying these changes early, practitioners can implement interventions that preserve healthy tissue and remineralize affected areas.

## Bacterial involvement:

Bacteria play a significant role in the progression of dental diseases, particularly in caries. Understanding the specific bacterial species and their metabolic products can inform treatment strategies. By utilizing techniques that address the bacterial component effectively, such as Silver Diamine Fluoride (SDF), dentists can halt disease progression without extensive removal of healthy tooth structure.

## Biomaterials:

The second component of the Golden Triangle focuses on the biomaterials used in restorative practices. The choice of materials greatly influences the effectiveness of minimally invasive techniques. Adhesive and Bonding Materials: Modern adhesive and bonding materials are essential for successful restorations in minimally invasive dentistry. These materials allow for effective adhesion to tooth structure while preserving maximum healthy tissue. Advances in bonding technology have led to materials that can bond to both dentin and enamel, enhancing the longevity and durability of restorations. Innovative Restorative Materials: Materials like bioactive glass, composite resins and glass ionomer cements not only restore tooth structure but also actively promote remineralization and protect against further decay. These materials are designed to mimic the natural properties of teeth, providing aesthetic and functional benefits.

## Clinical handling:

The final component of the Golden Triangle involves the clinical handling of materials and the patient throughout the treatment process.

## Material handling:

Proper handling and application of biomaterials are critical for achieving desired outcomes. This includes appropriate mixing, curing and placement techniques that ensure optimal bonding and minimize microleakage.

## Patient interaction:

Communication with the patient is also vital. Educating patients about the benefits of minimally invasive techniques helps build trust and encourages them to seek preventive care. A patient-centered approach also includes addressing their anxieties and ensuring comfort during procedures, which can significantly improve treatment outcomes.

## Minimal invasive restorative techniques:

Minimally invasive preventive strategies: Minimally invasive preventive strategies are essential in pediatric dentistry for assessing and managing a child's risk of dental caries. Evaluating this risk is crucial for creating personalized prevention plans tailored to individual risk factors [10]. A key model in this field is Caries Management by Risk Assessment (CAMBRA), which uses clinical evaluations, dietary assessments and fluoride exposure analysis to determine the best preventive and restorative interventions. This comprehensive approach helps dental professionals identify each child's unique risk profile [11]. CAMBRA-focused strategies encompass the use of targeted fluoride varnishes, the application of dental sealants, guidance on diet and modifications to follow-up schedules, all customized to meet each patient's specific needs. Studies indicate that interventions grounded in caries risk assessments can lower the incidence of cavities by 40-60% in children deemed high-risk over five years. This tailored approach not only boosts the efficacy of preventive measures but also supports lasting oral health for young patients [11, 12].

## Silver diamine fluoride:

Silver diamine fluoride marks a notable step forward in minimally invasive dentistry (MID). This topical solution harnesses the antibacterial power of silver along with the re-mineralizing benefits of fluoride, offering a comprehensive method to halt and prevent the progression of cavities. Its ease of use, affordability and non-invasive nature make it especially suitable for children, particularly those who are at high risk for cavities, have special needs, or struggle with dental anxiety [13]. SDF operates by creating a protective barrier of silver protein complexes that block bacterial activity, while fluoride ions promote the remineralization of enamel. Many studies have validated its ability to stop the progression of cavities, making it particularly valuable for young children and those with limited dental care access. Moreover, when applied to healthy tooth surfaces, SDF has demonstrated preventive benefits, helping to decrease the likelihood of new cavities forming [13]. In a recent systematic review and meta-analysis, Oliveira and colleagues found that SDF reduced the occurrence of dentin caries lesions in primary teeth by 77.5% compared to a placebo over 24 months or longer [14]. Additionally, research by Chu CH and his team showed that SDF was more effective than fluoride varnish at both 18 and 30 months, while glass ionomer cements performed better than SDF at the 12-month mark [15].

## Silver-modified atraumatic restorative treatment (SMART):

The Silver-Modified Atraumatic Restorative Treatment technique combines Silver Diamine Fluoride with restorative materials to effectively manage dental caries. After applying SDF, it seals the carious lesion, cutting off the nutrient supply to the decayed dentin and inhibiting bacterial survival. To minimize staining before placing a restorative material, potassium iodide (KI) is applied over the initial layer of SDF, enhancing acceptability for patients. Subsequently, a restoration, such as glass ionomer cement (GIC), is placed, which helps prevent the fracture of the remaining tooth structure, maintain space, facilitate biofilm removal and decrease the need for extensive behavior management during dental procedures [16, 17].

## Resin infiltration technique:

Resin infiltration is a gentle, non-invasive approach aimed at addressing early enamel caries and white spot lesions without the need for drilling or extensive preparation. This technique uses a low-viscosity resin that seeps into demineralized enamel, effectively filling in the porous areas and stabilizing the lesions to halt any further decay. While it was originally developed to tackle proximal caries, resin infiltration has gained considerable popularity in clinical practice for its success in managing non-cavitated lesions, especially among children and orthodontic patients. The process begins with the application of an etching agent, typically a 15% hydrochloric acid solution, which removes the outer hypermineralized layer of enamel, enhancing the resin's ability to penetrate deeper. Following this, a low-viscosity resin infiltrant is applied and cured using light polymerization, effectively sealing the lesion and preventing any additional demineralization. Table 1 provides a concise overview of the pros and cons associated with resin infiltration [18].

## Smart burs:

The traditional method for removing caries typically involves using various tools like spoon excavators, stainless steel round burs, diamond burs, or tungsten carbide burs at low speeds. One drawback of these techniques is that they can aggressively remove both infected and affected dentin. In contrast, a more conservative approach aims to specifically target and eliminate only the infected dentin, leaving affected dentin intact to allow for re-mineralization. Recently, a new self-limiting concept for mechanical caries removal has been introduced with the Polymer bur (SmartPrep, SS White Burs, Inc., Lakewood, NJ, USA). This paddle-shaped bur boasts a unique flute design and is crafted from medical-grade polyether-ketone-ketone (PEKK), with a knoop hardness of 50 KHN. These burs effectively remove soft carious dentin, but they dull upon contact with hard dentin, thus preventing unnecessary loss of the affected dentin [19]. In a study by Prabhakar A, the efficacy of polymer burs compared to carbide burs for caries removal was evaluated. The findings showed that polymer burs are self-limiting, losing their cutting efficiency upon reaching affected dentin and not cutting sound dentin. However, the time taken to remove caries with the polymer bur was significantly longer than with the carbon steel round bur [20].

## Bioactive restorative materials:

In the realm of minimal invasive dentistry, the significance of bioactive restorative materials cannot be overstated and Glass Ionomer Cement stands out as a key player. One of the primary advantages of GIC is its ability to release fluoride, a feature that plays a crucial role in preventing secondary caries and promoting the remineralization of demineralized dental structures. As GIC is placed, it gradually releases fluoride ions that help inhibit the growth of cariogenic bacteria and reinforce the integrity of surrounding enamel and dentin. This property makes GIC particularly advantageous in treating patients at high risk for caries, as it not only restores tooth function but also actively contributes to the long-term health of the tooth [21]. Over time, various modifications to traditional GIC have been developed to enhance its properties and performance in clinical settings. One notable modification is resin-modified glass ionomer cement (RMGIC), which integrates both glass ionomer and composite resin components. RMGICs offer improved aesthetic qualities and greater mechanical strength, making them suitable for a wider range of restorations while retaining essential fluoride release capabilities [21]. In addition to GIC, materials like Biodentine and Mineral Trioxide Aggregate (MTA) have emerged as important bioactive options in minimal invasive dentistry. Biodentine is particularly noteworthy as it serves as a dentin substitute, ideal for applications such as pulp capping or as a restorative material. This material mimics the physical properties of natural dentin and promotes healing by releasing calcium ions, which facilitate the formation of hydroxyapatite-a critical element for remineralization. While Biodentine lacks fluoride, its bioactive properties enhance the tooth structure and support healing processes effectively. MTA, on the other hand, is widely utilized in endodontic treatments, valued for its excellent sealing capabilities and biocompatibility, particularly in pulp capping and root-end filling procedures. MTA releases calcium ions that contribute to mineralization and facilitate the healing of periapical tissues, making it a strong choice in cases requiring bioactive restorative materials [12]. Recent advancements in nanotechnology have further revolutionized the field of dental materials, including GICs. The incorporation of nanoparticles has the potential to significantly enhance the mechanical properties, increase the rate of fluoride release and improve the aesthetic qualities of GICs. By exploiting the unique properties of nanoparticles, researchers can create next-generation GICs that are not only stronger and more durable but also preserve their bioactive functions. These innovations pave the way for more effective and reliable restorative options, further supporting the goals of minimal invasive dentistry [12].

## Chemo-mechanical caries removal approaches:

The conventional method of caries removal often results in excessive removal of healthy dentin due to a lack of tactile sensation, which can lead to pulp exposure. Additionally, the heat generated during the cutting process can negatively impact the pulp, causing inflammation and pain. The noise and vibration from handpieces may also trigger dental anxiety and discomfort. Removing both healthy and decayed dentin compromises the tooth structure and reduces its long-term durability [22, 23]. One of the most common minimally invasive dentistry (MID) modalities in the past decade has been the use of chemomechanical caries removal (CMCR) agents. CMCR involves chemically softening decayed dentin and then gently removing it with hand instruments. Unlike conventional surgical procedures, CMCR selectively removes infected dentin while preserving affected dentin that can potentially remineralize, making it a less destructive approach [22, 24]. Chemomechanical agents like sodium hypochlorite-based Carisolv and papain enzyme-based products such as Papacarie and Brix 3000 are used to selectively remove infected dentin in both primary and permanent teeth [4]. Healthy tissues contain alpha-1-antitrypsin, which prevents collagen breakdown by proteolytic enzymes. However, infected dentin lacks this protein, allowing the proteolytic enzymes in chemomechanical agents to degrade the collagen in infected areas, facilitating the selective removal of denatured collagen [25]. A systematic review and meta-analysis by Deng *et al.* comparing the effectiveness of Papacarie and conventional excavation in primary teeth found that Papacarie was effective for selectively removing carious tissue and was associated with significantly less pain compared to conventional excavation, although it required longer decay excavation times [26]. In a recent in vitro study by Santos *et al.* comparing the efficiency and efficacy of Papacarie, Brix 3000 and conventional excavation, it was concluded that while all methods effectively removed infected dentin, conventional excavation was more efficient, taking about 54 seconds, compared to Papacarie at 110.5 seconds and Brix 3000 at 85 seconds. However, conventional excavation was also associated with higher levels of pain [25].

## 3D printing technology:

3D printing technology is transforming minimal invasive dentistry by enabling precise customization and rapid production of dental appliances, crowns and models [27]. This innovative approach creates highly accurate restorations that fit patients' unique anatomies, significantly minimizing the need for extensive tooth reduction. Additionally, 3D printing facilitates the use of biocompatible materials, enhancing patient outcomes and comfort [28]. By streamlining workflows and reducing chair time, this technology improves efficiency in dental practices. Furthermore, 3D printing supports minimally invasive fabrication techniques, preserving healthy dental structures while delivering functional and aesthetic restorations [29]. Overall, it aligns perfectly with the principles of minimal invasive dentistry and modern patient-centered care [3].

## Advantages and limitation of minimal invasive restorative dentistry:

Table 2 provides an overview of the benefits and drawbacks of Minimal Invasive Restorative Dentistry.

## Future prospective of minimal invasive restorative dentistry:

The future of Minimal Invasive Restorative Dentistry is poised for exciting developments. As technology advances, we can expect enhanced diagnostic tools, innovative materials and more efficient techniques that will further reduce patient discomfort and improve outcomes [30]. Emphasizing preventive care, this approach will likely become the standard in dentistry, focusing on preserving healthy tooth structure. Additionally, education and training programs will be crucial in ensuring that dental professionals effectively implement these minimally invasive techniques. Overall, minimal invasive dentistry will continue to grow, prioritizing patient-centric care while promoting long-lasting oral health [31].

## Conclusion:

Minimal invasive restorative dentistry is transforming dental care by integrating advanced technology with a focus on prevention and preservation. This evolving field promises to improve patient experiences and outcomes through innovative techniques and materials. Thus, dental professionals can enhance care quality while prioritizing the preservation of natural teeth.

## Advancement to knowledge:

This review synthesizes current concepts and clinical techniques of minimal invasive restorative dentistry, emphasizing evidence-based caries management and conservative tooth preparation. It highlights advancements in adhesive and bioactive materials that support long-term tooth preservation and patient-centered care.

## Figures and Tables

**Table 1:** Overview of advantages and limitations of resin infiltration technique

**Advantages**	**Limitations**
Non-invasive treatment that preserves tooth structure.	Requires careful technique and effective moisture control.
Achievable in a single visit.	Not appropriate for cavities or dentin lesions.
Mechanically stabilizes demineralized enamel.	Potentially higher costs involved.
Allows deeper penetration into porous demineralized areas.	
Arrests or retards lesion progression.	
Minimizes the risk of secondary caries.	
No risk of postoperative sensitivity or pulpal inflammation.	
High patient acceptance.	

**Table 2:** Advantages and limitation of minimal invasive restorative dentistry

**Advantages**	**Limitation**
Preservation of healthy tooth structure.	Limited applicability for extensive decay
Reduced patient discomfort and anxiety.	Higher initial costs for advanced materials.
Enhanced aesthetics of restorations.	Increased reliance on technology.
Lower risk of pulp exposure and complications.	Limited restoration options for severe tooth damage.
Improved longevity of restorations.	
More conservative treatment options.	
Greater treatment flexibility.	
Use of biocompatible materials.	
